# A primary malignant fibrous histiocytoma of the scalp and intracranial tumor bleeding: a case report

**DOI:** 10.1186/1752-1947-8-50

**Published:** 2014-02-13

**Authors:** Jie Wang, Weiming Zhong, Yinghui Xu, Le Feng, Yang Li, Bin Dong

**Affiliations:** 1 Department of Neurosurgery, First Affiliated Hospital of Dalian Medical University, 222 Zhongshan Road, Xigang District, Dalian, China

**Keywords:** Malignant fibrous histiocytoma, Sarcoma, Scalp

## Abstract

**Introduction:**

A malignant fibrous histiocytoma occurring on the scalp near a primary operation site is extremely rare.

**Case presentation:**

A 74-year-old Chinese man presented with a one-month history of recurrent headaches, vomiting and left limb atony. He had undergone a successful clipping operation through the pterional approach for an anterior communicating aneurysm five years previously. One month before presentation, he developed a headache without apparent cause that was especially severe in the right frontal region. He also had a small tumor on the right side of his forehead at the original incision site. The tumor had gradually increased from soybean size to egg size in one month; this growth was accompanied by nausea, projectile vomiting of gastric contents and left limb atony. The subcutaneous tumor was totally resected along with some affected cranial tissues. Our patient’s postoperative recovery was good, and he was safely discharged 20 days after the surgery. He was free of recurrence over two years of follow-up.

**Conclusions:**

Dissection of the temporal muscles and deep fascia using electric resection and electrocoagulation through the pterional approach may cause tissue degeneration, which may in turn lead to cancer development. In our patient’s case, the reason for the development of the tumor five years after his surgical aneurysm repair was unclear; it may have represented a primary malignant fibrous histiocytoma of the scalp that had no relationship to the operation. We followed up our patient for two years and he had no tumor recurrence. Because malignant fibrous histiocytoma of the scalp has a high degree of malignancy and readily recurs *in situ*, early diagnosis and radical surgical resection are key to a successful outcome.

## Introduction

First reported by O’Brien and Stout in 1964 [1] a malignant fibrous histiocytoma (MFH) is a soft tissue neoplasm with a poor prognosis. It often occurs in the deep soft tissues, such as the fascia and fibrous tissue. In 1972, Feldman and Norman [2] stated that this tumor is a separate type of bone tumor. A MFH that occurs on the scalp near a primary operation site is extremely rare, and one that is accompanied by intracranial stroke is even rarer. We report the rare case of a patient with an MFH that occurred on the right side of his forehead at an original operation site. The tumor grew rapidly and eventually led to intracranial hemorrhage.

## Case presentation

**Figure 1:**
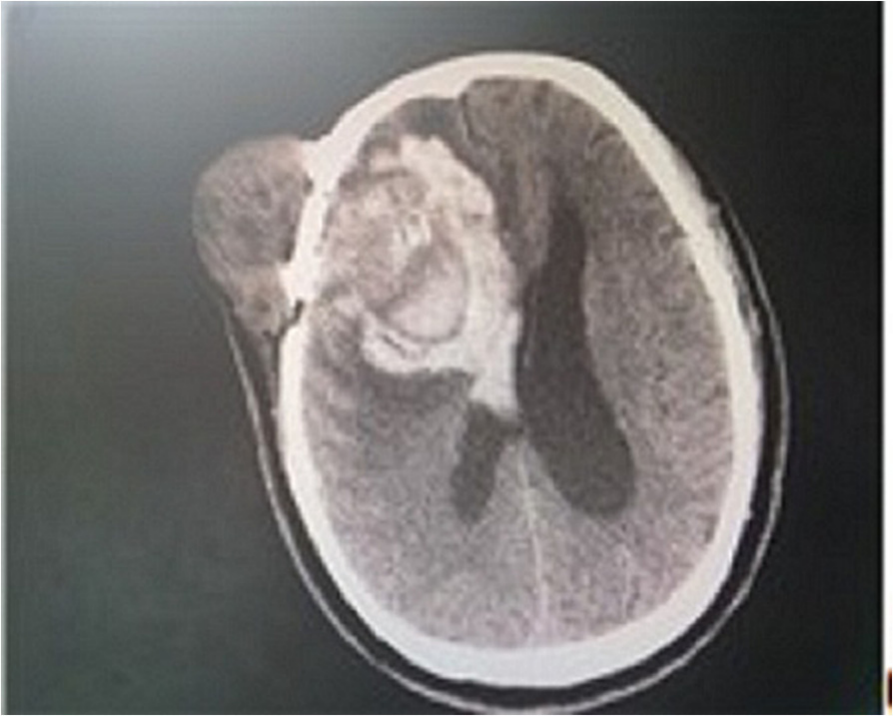
**Preoperative computed tomography.** Shows intracranial hemorrhage with a volume of approximately 90mL, and a midline shift of over 1.5cm.

A 74-year-old Chinese man presented with a one-month history of recurrent headaches, vomiting and left limb atony. He had undergone a successful clipping operation through the pterional approach for an anterior communicating artery aneurysm five years previously. One month before presentation, he developed a headache without apparent cause that was especially severe in the right frontal region. He also had a small tumor on the right side of his forehead at the original incision site; the tumor had gradually increased from soybean size to egg size in one month. This was accompanied by nausea, projectile vomiting of gastric contents and left limb atony. Computed tomography demonstrated a subcutaneous tumor on the right side of his forehead, a scalp lesion complicated with right frontal lobe hemorrhage, and a right ventricular hematocele. We gave our patient oxiracetam (5.0g in 250mL of normal saline (0.9% weight to volume of sodium chloride)) for treatment of neurotrophy. On the third day after admission, he suddenly lost consciousness. Urgent head computed tomography showed an intracranial hemorrhage, the volume of which had increased from 40mL to 90mL as well as persistent bleeding and a midline shift of over 1.5cm (Figure 1).

**Figure 2:**
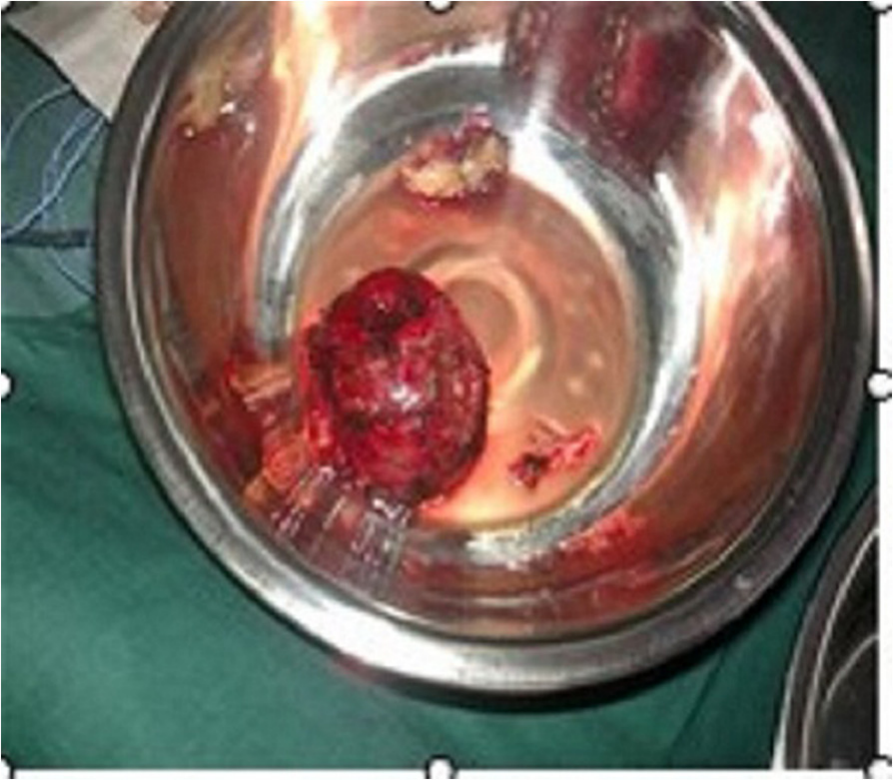
**Intraoperative invasion.** Tumor invasion of the skull was noted during the surgery.

**Figure 3:**
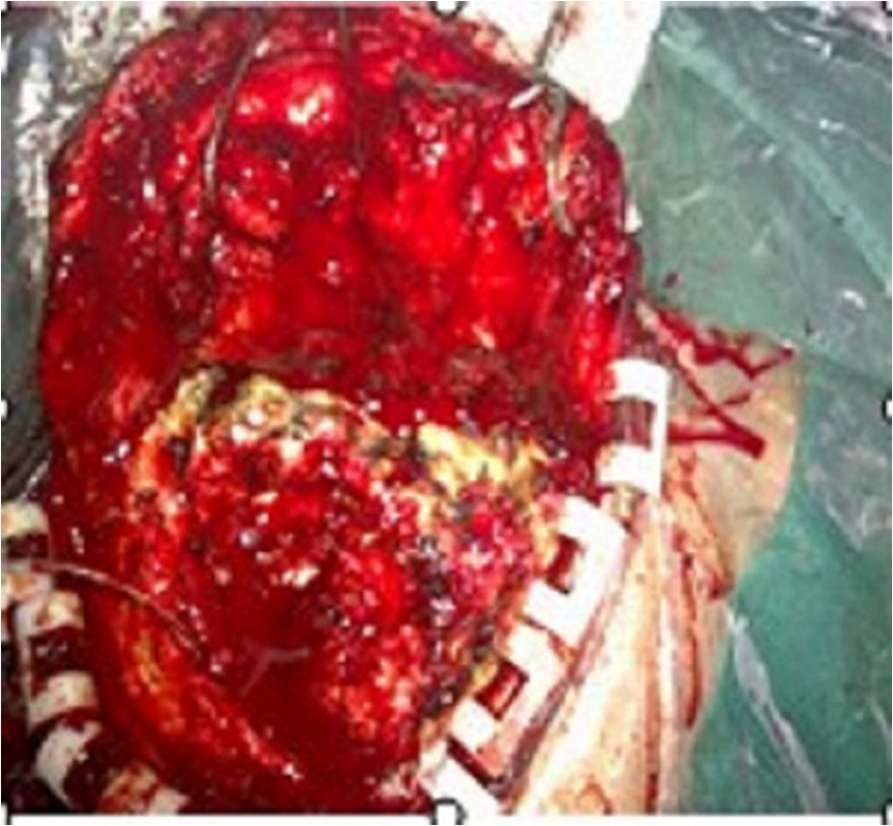
**Tumor resection.** The tumor was completely resected and measured 7cm×6cm×4cm.

Our patient then underwent surgery to resect the subcutaneous tumor and evacuate the intracranial hematoma under general anesthesia. An incision was made along the one from the original surgery. During the operation, it became apparent that the main part of the tumor was located in the subcutaneous tissue of his right frontal region. The tumor was approximately 7cm×6cm×4cm (Figure 2), fully enveloped, and characterized by abundant revascularization. The cyst of the tumor was intact, and his temporal muscle tissue was thin. His cranial periosteum and cerebral dura mater were invaded by the tumor, resulting in a thin, brittle skull (Figure 3). His dura mater was also thin and light yellow in color. The tumorous hemorrhage extended into his intracranial tissue, but no tumor tissue was seen in his subdural area. The intracranial hematoma was successfully removed during the operation (70mL from the right frontal part, 20mL from the intraventricular hematoma). The subcutaneous tumor was totally resected along with some affected cranial tissues. The epidural bleeding was completely stopped. However, the temporal muscle fascia was incomplete because of the hemostasis. An artificial dura was created by relaxation sutures to prevent cerebrospinal fluid leakage. His residual temporal muscle was then sutured, and his galea aponeurosis and scalp were perfectly closed. Our patient’s postoperative recovery was good, and he was safely discharged 20 days after the surgery. He was free of recurrence over two years of follow-up.

**Figure 4:**
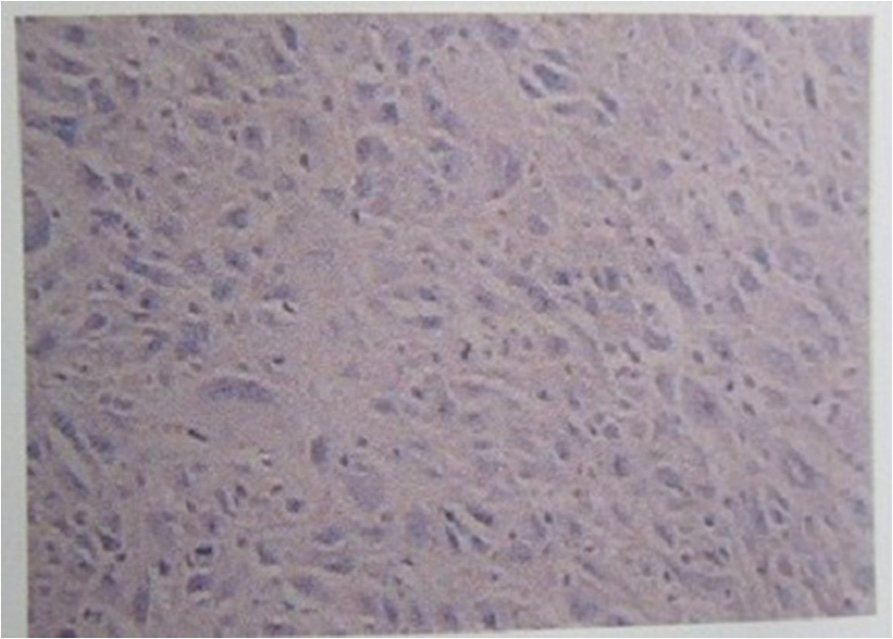
**Postoperative pathological findings.** Immunohistochemical staining ×100 magnification.

A pathological microscopic examination of the resected tumor (Figure 4) showed a malignant histiocytoma (undifferentiated sarcoma). It was gray-white, had no capsule, was gray on the cut section, had areas of hemorrhage and necrosis, and had a ductile texture. Microscopic examination showed pleomorphic tumor cells, specifically fibroblasts, histiocytes, multinucleated cells and some inflammatory cells infiltration, as shown in Figure 4. Immunohistochemistry results showed it was negative for keratin, S-100 protein, actin, desmin, homatropine methylbromide 45, cluster of differentiation 34 and b-cell lymphoma 2, and positive for vimentin and cluster of differentiation 99, with Ki-67 content over 60%.

## Discussion

MFH is one of the most common soft tissue sarcomas in adults [3,4]. The most common sites of origin are the proximal extremities, particularly the thigh and buttock [4]. This tumor presents as a multilobular fleshy mass, often apparently circumscribed, although the microscopic growth pattern is frequently infiltrative among fascial planes and between muscle fibers, accounting for its high rate of local recurrence [5]. Four established subtypes have been described, each with similar prognostic features. The most common variant is the storiform pleomorphic type, which comprises spindle cells in a storiform pattern, plump histiocyte-like cells and pleomorphic multinucleated giant cells [6,7]. The prognosis of MFH is generally poor [8]. Definitive treatment is surgical, either with wide local excision or total resection. An MFH occurring on the scalp is extremely rare, and one accompanied by intracranial stroke is even rarer [9,10]. In our case, our patient was an elderly man with no immediate relevant medical history and no prior radiation exposure, but he did have a history of an anterior communicating artery aneurysm clipping operation. A subcutaneous tumor was seen on the right side of his forehead at the original incision site. Dissection of his temporal muscle tissue and deep fascia using electric resection and electrocoagulation through the pterional approach may have resulted in tissue degeneration, which in turn might have led to the cancer. The reason for the development of this tumor five years after the aneurysm operation was unclear. It is possible that the tumor was a primary MFH of the scalp. Our patient was followed-up for two years without recurrence. Because MFH of the scalp has a high degree of malignancy and easily recurs *in situ*, early diagnosis and radical surgical resection are key to a successful outcome [11,12].

## Conclusions

The cancer in our patient may have been induced by dissection of his temporal muscle tissue and deep fascia using electric resection and electrocoagulation through the pterional approach. MFH of the scalp has a high degree of malignancy and recurrence *in situ*, thus early diagnosis and radical surgical resection are key to a successful outcome.

## Consent

Written informed consent was obtained from the patient for publication of this case report and accompanying images. A copy of the written consent is available for review by the Editor-in-Chief of this journal.

## Competing interests

The authors declare that they have no competing interests.

## Authors’ contributions

WJ analyzed and interpreted the patient data and searched the existing literature on malignant fibrous histiocytoma. XYH and FL obtained the patient’s photographs and designed the figures. DB wrote the case report and revised the manuscript. LY and ZWM assisted with the writing and editing of the manuscript. All authors read and approved the final manuscript.
